# Age and Gender Impact the Measurement of Myocardial Interstitial Fibrosis in a Healthy Adult Chinese Population: A Cardiac Magnetic Resonance Study

**DOI:** 10.3389/fphys.2018.00140

**Published:** 2018-03-06

**Authors:** Yang Dong, Dan Yang, Yuchi Han, Wei Cheng, Jiayu Sun, Ke Wan, Hong Liu, Andreas Greiser, Xiaoyue Zhou, Yucheng Chen

**Affiliations:** ^1^Cardiology Division, West China Hospital, Sichuan University, Chengdu, China; ^2^Cardiovascular Division, Department of Medicine, University of Pennsylvania, Philadelphia, PA, United States; ^3^Radiology Department, West China Hospital, Sichuan University, Chengdu, China; ^4^Siemens Healthcare GmbH, Erlangen, Germany; ^5^Northeast Asia MR Collaboration, Siemens Healthcare, Beijing, China

**Keywords:** myocardial interstitial fibrosis, T1 mapping, native T1, extracellular volume (ECV), gender, age, Chinese population

## Abstract

**Background:** Diffuse myocardial fibrosis is a common pathological process in many cardiovascular diseases. In order to determine disease, we must have standard normal imaging values. We investigated myocardial interstitial fibrosis of the left ventricle (LV) in a healthy population of Chinese adults and explored the impact of gender, age, and other physiological factors using a T1 mapping technique of cardiac magnetic resonance imaging (CMR).

**Materials and Methods:** We recruited 69 healthy adult Chinese subjects (35 males; age 18–76). LV function and global strain were obtained from functional imaging. T1 mapping was performed using a modified look-locker sequence. Global and segmental native T1 and extracellular volume (ECV) were calculated using dedicated software. Gender, age, and segmental variation of both native myocardial T1 and ECV of the LV were analyzed.

**Results:** The global myocardial native T1 and ECV of the LV in this Chinese adult healthy population was 1,202 ± 45 ms and 27 ± 3% at 3T field strength, respectively. Females had a higher myocardial native T1 and ECV of the LV compared to males [1,210 (1,188–1,264) ms vs. 1,182 (1,150–1,211) ms, *P* < 0.001; 28 ± 3 vs. 26 ± 3%, *P* = 0.027, respectively]. ECV in older group was higher than younger group [27 (26–29)% vs. 25 (24–29), *P* = 0.019]. The multi-variate linear regression analysis showed that only gender (Beta = −0.512, *P* < 0.001) was independently related with global native T1 of LV while gender (Beta = −0.278, *P* = 0.017) and age (Beta = 0.303, *P* = 0.010) were independently related with global ECV of LV. From the base to apex of the LV, myocardial native T1 (*P* = 0.020) and ECV (*P* < 0.001) significantly increased. Within the same slice of the LV, there were significant segmental variations of both myocardial native T1 (*P* < 0.001) and ECV (*P* < 0.001) values.

**Conclusion:** Gender and age have significant impacts on the imaging markers of myocardial interstitial fibrosis in healthy adult Chinese volunteers. Segmental variation of myocardial interstitial fibrosis was also observed.

## Introduction

The myocardium is composed of myocytes and many different cell types including the fibrocytes that occupy the interstitium. Myocardial fibrosis is the remodeling of extracellular matrix in response to injury and is a common pathological process in many cardiovascular diseases (Murtha et al., 2017). The fibrogenic growth factors, such as transforming growth factor-β and platelet derived growth factor, and angiotensin/aldosterone axis have been proven to be involved in myocardial fibrosis process (Kong et al., 2014; Murtha et al., 2017). Some researchers have pointed out that in aging, myocardial fibrosis appears as diffuse fibrosis (Horn and Trafford, 2016). The cardiac magnetic resonance (CMR) T1 mapping technique is emerging as a robust and reproducible technique to quantify myocardial fibrosis by estimating the myocardial T1 value (Salerno and Kramer, 2013; van Oorschot et al., 2015). Previous histopathological studies have demonstrated good correlation between extracelluar volume (ECV) and myocardial interstitial fibrosis in animals and humans (Neilan et al., 2013; aus dem Siepen et al., 2015). Therefore, the T1 mapping technique provides a direct, non-invasive method to evaluate myocardial interstitial fibrosis, which cannot be assessed by the CMR late gadolinium enhancement (LGE) technique.

Age and gender difference in cardiovascular disease is an interesting phenomenon. There is an increased incidence of diastolic heart failure with aging (Fischer et al., 2003; Owan et al., 2006). Cardiac diastolic function is closely related to stiffness caused by myocardial interstitial fibrosis (Moreo et al., 2009). It is important to explore the relationship of gender and age to myocardial interstitial fibrosis, so as to aid understanding of the pathophysiology of diastolic heart failure that is prevalent in the elderly, particularly women. In previous studies, the T1 mapping technique was used to demonstrate the impact of gender and age on myocardial fibrosis in healthy populations (Piechnik et al., 2013; Puntmann et al., 2013; von Knobelsdorff-Brenkenhoff et al., 2013; Dabir et al., 2014; Liu et al., 2014; Kawel-Boehm et al., 2015; Rauhalammi et al., 2016), but the results were conflicting. Furthermore, no previous studies had been performed in the Chinese population. Finally, study of regional cardiac lesions such as infarction, inflammation, and remodeling, requires a segment-based analysis of myocardial fibrosis. Therefore, the present study aims to investigate the impact of age and gender on T1 and ECV values, as well as segmental variation in the left ventricle (LV) in a healthy adult Chinese population.

## Materials and methods

The study protocol was approved by the Ethics Committee of West China Hospital. All participants provided written informed consent.

### Study subjects

A total of 69 (35 males) healthy Chinese volunteers age ≥18 years were prospectively recruited between 2014 and 2016. Exclusion criteria were: any known cardiovascular disease, hypertension, cerebrovascular disease, neurologic disease, chronic lung disease, diabetes, cancer, autoimmune disease, recent systemic infection (within a month), recent operation or severe trauma (within a month), any recent medications, or history of pacemaker or other metallic object in the body (a contraindication for magnetic resonance imaging). Subjects also were excluded for: a resting blood pressure equal to or >140/90 mmHg, abnormal complete blood count, abnormal liver and renal function tests, abnormal ECG, abnormal findings by echocardiography, incidental arrhythmias, claustrophobia, intolerance to breath-holding during scanning and poor image quality, any LGE found following CMR examination.

### Imaging acquisition

Data acquisition was performed on a 3.0T MRI scanner (Magnetom Tim Trio; Siemens Medical Solutions, Erlangen, Germany) using a four-channel phased-array receiver coil combined with a four-channel spine coil. Images were acquired during breath-holds with retrospective ECG gating. Steady-state free precession (SSFP) cine images were acquired in consecutive short-axis views covering the LV from base to apex and three long-axis views (two-, three-, and four-chamber). The parameters were as follows: repetition time (TR), 3.4 ms; echo time (TE), 1.3 ms; flip angle (FA), 50°; field of view (FOV), 320–340 mm; matrix size, 256 × 144; and slice thickness, 8 mm with no gap. Temporal resolution was around 42 ms. Reconstruction in plane spatial resolution was 1.4 × 1.3 mm.

Native T1 measurements were acquired before injection of gadolinium by the motion-corrected modified Look-Locker inversion recovery sequence (MOLLI) [Siemens healthcare works-in-progress 448; protocol: 5(3)3]. Measurements were acquired at the basal, mid, and apical slices on short axis (SAX) through ECG gating. This involved three inversion-recovery prepared inversion time (TI) scout experiments, with three heartbeats for recovery between each experiment. Parameters for MOLLI were as follows: TR 2.9 ms, TE 1.12 ms, FA 35°, bandwidth 930 Hz/pixel, TI of first experiment 100 ms, TI increment 80 ms, parallel imaging 2, matrix 192 × 144, in plane spatial resolution 2.4 × 1.8 mm, total acquisition time 17 heart beats. T1 measurements were repeated in the identical short-axis slices 15 min following administration of gadolinium [MOLLI protocol: 4(1)3(1)2]. Gadopentetate dimeglumine (Magnevist, Bayer HealthCare Pharmaceuticals, Wayne, New Jersey, USA) was used with a bolus dose of 0.15 mmol/kg. Hematocrit (HCT) was tested within 24 h of Cardiac MR scanning for ECV calculation.

### Post-procession analysis

#### Cardiac dimension, volume, and function

All CMR images were post-processed using dedicated Cardiac MR post-procession software (Qmass 7.6, Medis, Leiden, the Netherland) by two trained observers with more than 2 years and 500-case experience in CMR image analysis. LV volume and mass were measured by manually tracing endocardial and epicardial borders at end-diastole and end-systole on successive short-axis cine images. End-diastolic volume (EDV) and end-systolic volume (ESV) were calculated by summation of the volume in consecutive short-axis slices. Ventricular stroke volume (SV) was formulated as: SV = EDV – ESV. Ventricular ejection fraction (EF) was calculated as: EF = SV/EDV. LV mass was derived by the summation of discs method by multiplying myocardial muscle volume (epicardial contour area – endocardial contour area) by its specific density (1.05 g/cm^3^). Papillary muscles were excluded in the LV mass calculation. Left atrial maximal volume (LAVmax) was analyzed according to bi-plane Simpson method based on four- and two-chamber SSFP cine images, according to our previous report (Li et al., 2017).

#### Feature-tracking MRI analysis for left ventricular myocardial strain

Cine SSFP-based peak systolic strain measurements were analyzed by feature tracking (FT) analysis (Medis Suite 2.1, Medis, Leiden, The Netherlands). Radial or circumferential strain was quantified on short-axis view at mid-level of the LV. Longitudinal strain was quantified on a standard four-chamber view. LV endocardial and epicardial contours were drawn manually at end-diastole and consecutive contours at the other phases through the cardiac cycle were tracked automatically by the FT algorithm. Peak global systolic radial, longitudinal, and circumferential myocardial strains were included in the analysis.

#### Native T1 and ECV analysis

Native T1 calculation was based on the MOLLI images in the three LV short-axis slices. Endocardial and epicardial contours were traced manually on the pre- and post-contrast T1-mapping images. After fitting the T1 curve (QMass 7.6, Medis, Leiden, The Netherlands), mean myocardial native T1 values were obtained. Blood T1 was obtained by locating a region of interest (ROI) in the blood pool within the LV cavity (avoiding papillary muscle) in the pre- and post-contrast T1-mapping images. ECV was calculated as follows: ECV = (1-HCT) × ([1/T1myocardial post-contrast – 1/T1myocardial pre-contrast]/[1/T1blood post-contrast – 1/T1blood pre-contrast]) (de Meester de Ravenstein et al., 2015). We performed segmental analysis according to the American Heart Association (AHA) 16-segment model (Cerqueira et al., 2002).

### Inter- and intra-observer variability

Fourteen cases (20% of total subjects) were randomly selected from the cohort to test observer variability. For inter-observer variability, the two independent observers performed post-processing, blinded to each other's results. For intra-observer variability, one observer repeated the measurements using the identical method 8 weeks later.

### Statistical analysis

Statistical analyses were performed using MedCalc (MedCalc Software version 13.0; Ostend, Belgium) and GraphPad Prism version 6.0 (GraphPad Software, San Diego, CA, USA). The Kolmogorov–Smirnov test was employed to check normal distribution of continuous variables. Continuous data are presented as mean ± *SD* and the range from minimum to maximum values if the distribution was normal while we presented the data not normally distributed as median and interquartile range. Differences in continuous variables between two groups were analyzed using the Student's *t-*test if the distribution was normal. If the distribution was not normal, the differences were tested with Mann–Whitney *U*-test. Comparisons in continuous variables between three or more groups were analyzed using a one-way analysis of variance (ANOVA) if the distribution was normal while the Kruskal–Wallis test was used if the distribution was not normal. No adjustments were made for multiple testing. Differences in categorical variables between two groups were analyzed using the chi-square test. Linear regression was used for analysis of relationships between the parameters (native T1 and ECV) and physiological variables. Multiple linear regression was used to test the relationship between parameters (native T1 and ECV) and demongraphic and CMR parameters. Inter- and intra-observer variability were tested by calculating mean bias from Bland–Altman analyses, coefficient of variation (COV, %), and inter-class correlation coefficient (ICC). *P* < 0.05 was considered statistically significant.

## Results

### Demographic data

Demographic data for the 69 subjects are displayed in Table 1. There was no age difference between the male and female groups. Body mass index (BMI) and hematocrit were higher in the male group. BMI was also higher in older group. No significant differences in blood pressure and heart rate were observed between groups.

**Table 1:** Demographic characteristics of a cohort of healthy adult Chinese volunteers.

	**Total (*n* = 69)**	**Male (*n* = 35)**	**Female (*n* = 34)**	***P*-value (M vs. F)**	**Younger group (*n* = 35)**	**Older group (*n* = 34)**	***P*-value (Y vs. O)**
Male (%)	51	–	–	–	51	50	0.904
Age (years)	46 ± 16 (18–76)	47 ± 18 (18–76)	46 ± 14 (18-69)	0.782	28 ± 10 (18–45)	59 ± 8 (46–76)	<0.001
BMI (kg/m2)	22 ± 3 (18–29)	23 ± 3 (18–29)	22 ± 3 (18–29)	0.038	22 ± 2 (18–27)	23 ± 3 (19–29)	0.018
HR (bpm)	75 (67–80)	73 (66–80)	78 (73–82)	0.115	77 (68–80)	75 (67–80)	0.474
SBP (mmHg)	122 ± 10 (102–139)	123 ± 9 (105–139)	121 ± 10 (102–139)	0.387	120 ± 9 (102–139)	124 ± 10 (105–139)	0.075
DBP (mmHg)	74 (70–80)	76 ± 7 (60–89)	73 ± 9(60–89)	0.172	72 (69–79)	75 (70–81)	0.128
HCT (%)	42 (40–45)	45 (43–46)	41 (40–42)	<0.001	44 ± 4 (37–50)	42 ± 3 (38–53)	0.177

*M, male; F, female; Y, younger group (≤45 years old); O, older group (>45 years old); BMI, body mass index; HR, heart rate; SBP, systolic blood pressure; DBP, diastolic blood pressure; HCT, hematocrit. Continuous data are presented as mean ± SD and the range from minimum to maximum values if the distribution was normal while we presented the data not normally distributed as median and interquartile range*.

### Left ventricular volume, function, and myocardial strain parameters of CMR

LV volume, systolic function, and strain parameters measured by FT-CMR are displayed in Table 2. LV mass index and LV end-diastolic volume index (EDVI) were greater in the male group. No gender differences were found in LV end systolic volume index (ESVI) and LVEF. LVEDVI and LVESVI were higher while LVEF was lower in the younger (≤45 years; *n* = 35) group compared with the older group (>45 years; *n* = 34). Females had higher global strains than males, including radial, circumferential, and longitudinal, while older group had higher radial strain. Indexed LAVmax showed no significant difference between age or gender subgroups.

**Table 2:** CMR parameters of a cohort of healthy adult Chinese volunteers.

	**Total (*n* = 69)**	**Male (*n* = 35)**	**Female (*n* = 34)**	***P*-value (M vs. F)**	**Younger group (*n* = 35)**	**Older group (*n* = 34)**	***P*-value (Y vs. O)**
LVEDVI (ml/m^2^)	78 ± 12 (44–106)	81 ± 11 (64–106)	75 ± 13 (44–105)	0.034	82 ± 13 (64–106)	73 ± 10 (44–94)	0.002
LVESVI (ml/m^2^)	29 ± 7 (11–47)	31 ± 8 (18–47)	28 ± 6 (11–46)	0.114	32 ± 7 (21–47)	26 ± 6 (11–40)	<0.001
LVEF (%)	61 (59–66)	62 ± 5 (55–74)	63 ± 4 (56–75)	0.794	60 (59–65)	64 (60–68)	0.024
LVmassi (g/m^2^)	46 ± 10 (26–72)	50 ± 10 (28–72)	42 ± 7 (26–54)	<0.001	45 (41–54)	46 (38–51)	0.361
GLS (%)	−23.9 ± 4.7 [(−35.9)–(−16.2)]	−21.8 [(−24.9)–(−20.3)]	−25.8 [(−27.1)–(−22.9)]	0.010	−23.2 [(−26.4)–(−21.7)]	−24.4 [(−27.4)–(−20.3)]	0.810
GCS (%)	−23.3 ± 3.7 [(−32.8)–(−15.4)]	−21.8 [(−25.2)–(−18.9)]	−24.2 [(−26.3)–(−21.7)]	0.027	−23.0 ± 3.3 [(−29.5)–(−18.4)]	−23.5 ± 4.0 [(−32.8)–(−15.4)]	0.574
GRS (%)	92 ± 40 (23.7–169.8)	75.2 (61.0–91.9)	109.3 (86.3–126.2)	0.001	75.0 (61.0–94.0)	109.3 (78.3–122.0)	0.012
LAVmaxi (ml/m^2^)	40.4 ± 8.0 (20.6–59.3)	39.6 ± 8.6 (20.6–58.9)	41.2 ± 7.4 (26.1–59.3)	0.420	40.3 ± 8.1 (20.6–53.6)	40.5 ± 8.0 (26.2–59.3)	0.903
Native T1 (ms)	1,202 ± 45 (1,099–1,300)	1,182 (1,150–1,211)	1,210 (1,188–1,264)	<0.001	1,198 ± 43 (1,110–1,285)	1,206 ± 47 (1,099–1,300)	0.466
ECV (%)	27 ± 3 (22–34)	26 ± 3 (22–33)	28 ± 3 (22–34)	0.027	25 (24–29)	27 (26–29)	0.019

*M, male; F, female; Y, younger group (≤45 years old); O, older group (>45 years old); LV, left ventricle; EDVI, end-diastolic volume index; ESViI, end-systolic volume index; EF, ejection fraction; massi, mass index; ECV, extracelluar volume; GLS, global longitudinal strain; GCS, global circumferential strain; GRS, global radial strain; LAVmaxi, left atrium maximal volume index. Continuous data are presented as mean ± SD and the range from minimum to maximum values if the distribution was normal while we presented the data not normally distributed as median and interquartile range*.

### Myocardial native T1 value, ECV, and differences in different age and gender groups

A total of 1,046 (94.7%) segments in 69 subjects were analyzed. Seven basal slices (two from males and five from females) and four apical slices (three from males and one from females) were excluded due to artifacts or poor image quality. Native T1 values and ECV are shown in Table 2. Both native T1 value (*P* < 0.001) and ECV (*P* = 0.027) was significantly higher in female group. There was no significant difference in myocardial native T1 value between older and younger groups, while ECV in older group was higher than younger group (*P* = 0.019). By linear regression, only gender was independently related with native T1 (*P* < 0.01). Besides, only age and gender were independently related with ECV (age: *P* = 0.01; gender: *P* = 0.017; Table 3).

**Table 3:** Linear regression analysis of global native T1 and ECV.

	**Native T1 (ms)**				**ECV (%)**			
	**Univariate**		**Multivariate**		**Univariate**		**Multivariate**	
			**(R**^**∧**^**2** = **0.262**, ***P*** < **0.001)**				**(R**^**∧**^**2** = **0.162**, ***P*** = **0.003)**	
	**Beta**	***P***	**Beta**	***P***	**Beta**	***P***	**Beta**	***P***
Age (years)	0.053	0.666	–	–	0.287	0.017	0.303	0.010
Gender^Δ^	−0.512	<0.001	-0.512	<0.001	−0.267	0.027	−0.278	0.017
BMI (kg/m^2^)	−0.123	0.312	–	–	−0.067	0.586	–	–
SBP (mmHg)	0.011	0.929	–	–	−0.161	0.187	–	–
DBP (mmHg)	−0.064	0.602	–	–	−0.033	0.787	–	–
HR (min^−1^)	0.052	0.671	–	–	0.074	0.546	–	–
LVEF (%)	−0.011	0.932	–	–	0.197	0.106	–	–

Δ *, For gender, we used 0 to represent for female and 1 to represent for male; ECV, extracelluar volume; BMI, body mass index; SBP, systolic blood pressure; DBP, diastolic blood pressure; HR, heart rate; LVEF, left ventricle ejection fraction*.

### Association between myocardial native T1/ECV and cardiac functional parameters

No significant correlation was found between either myocardial native T1 or ECV and cardiac functional parameters(LVEDVI, LVESVI, LVEF, and LV mass index; all *P* > 0.1). Furthermore, no correlation was found between either native T1 or ECV and LV strain (global longitudinal strain, global circumferential strain, and global radial strain; all *P* > 0.05). In addition, no association was found between LAVmaxi and myocardial native T1 or ECV (all *P* > 0.1).

### Segmental myocardial native T1 and ECV of LV

Values of myocardial native T1 and ECV in basal, mid, and apical slices of LV are shown in Table 4. There was a trend of increasing T1 value and ECV from the basal to apical slice. Gender-specific data showed the same trend with the exception of the T1 value in males (*P* = 0.342; Table 4).

**Table 4:** Myocardial native T1 and ECV at difference slices.

		**Total (*n* = 69)**	**Male (*n* = 35)**	**Female (*n* = 34)**	***P*-value (M vs. F)**
Native T1 (ms)	Basal	1,197 ± 46 (1,114–1,311)	1,164 (1,150–1,200)	1,217 (1,190–1,252)	<0.001
	Mid	1,202 ± 45 (1,099–1,300)	1,182 (1,150–1,211)	1,210 (1,188–1,264)	<0.001
	Apical	1,229 ± 70 (1,079–1,405)^*^	1,192 ± 54 (1,079–1,343)	1,265 ± 66 (1,141–1,405)^*^	<0.001
	Basal	26 ± 3 (20–33)	25 ± 3 (20–32)	27 ± 3 (21–33)	0.029
ECV (%)	Mid	27 ± 3 (22–34)	26 ± 3 (22–33)	28 ± 3 (22–34)	0.027
	Apical	29 (27–32)^*^	28 (25–31)^*^	31 (28–33)^*^	0.006

ECV, extracelluar volume;

* *, significant differences between different slices (P < 0.05 by ANOVA or Kruskal–Wallis test). Continuous data are presented as mean ± SD and the range from minimum to maximum values if the distribution was normal while we presented the data not normally distributed as median and interquartile range*.

**Figure 1:**
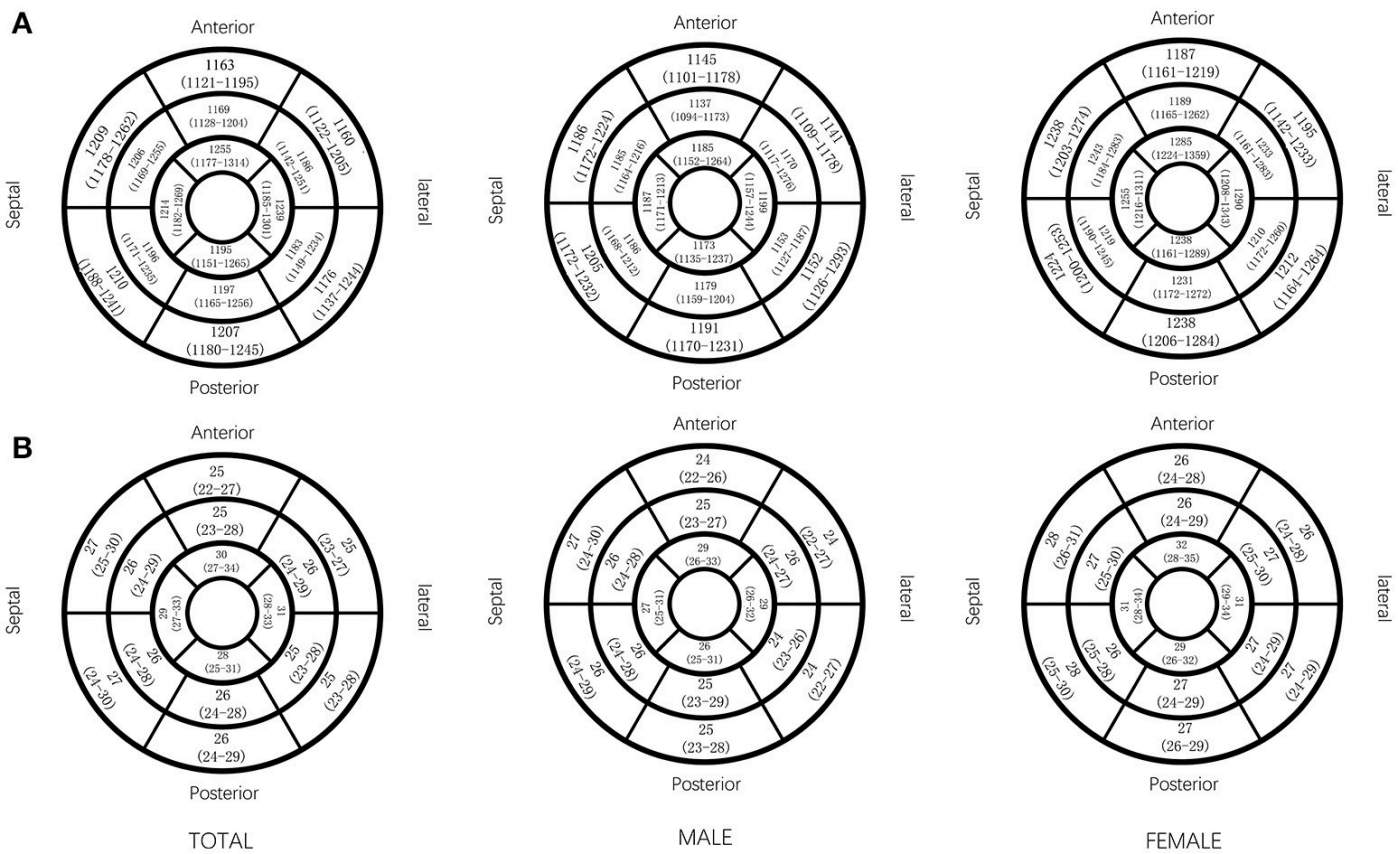
Native T1 value and ECV (median and interquartile range) in AHA 16 segments displayed by bull's eye model, by gender. Native T1 (ms) value **(A)** and ECV (%) value **(B)** in AHA 16 segments.

**Figure 2:**
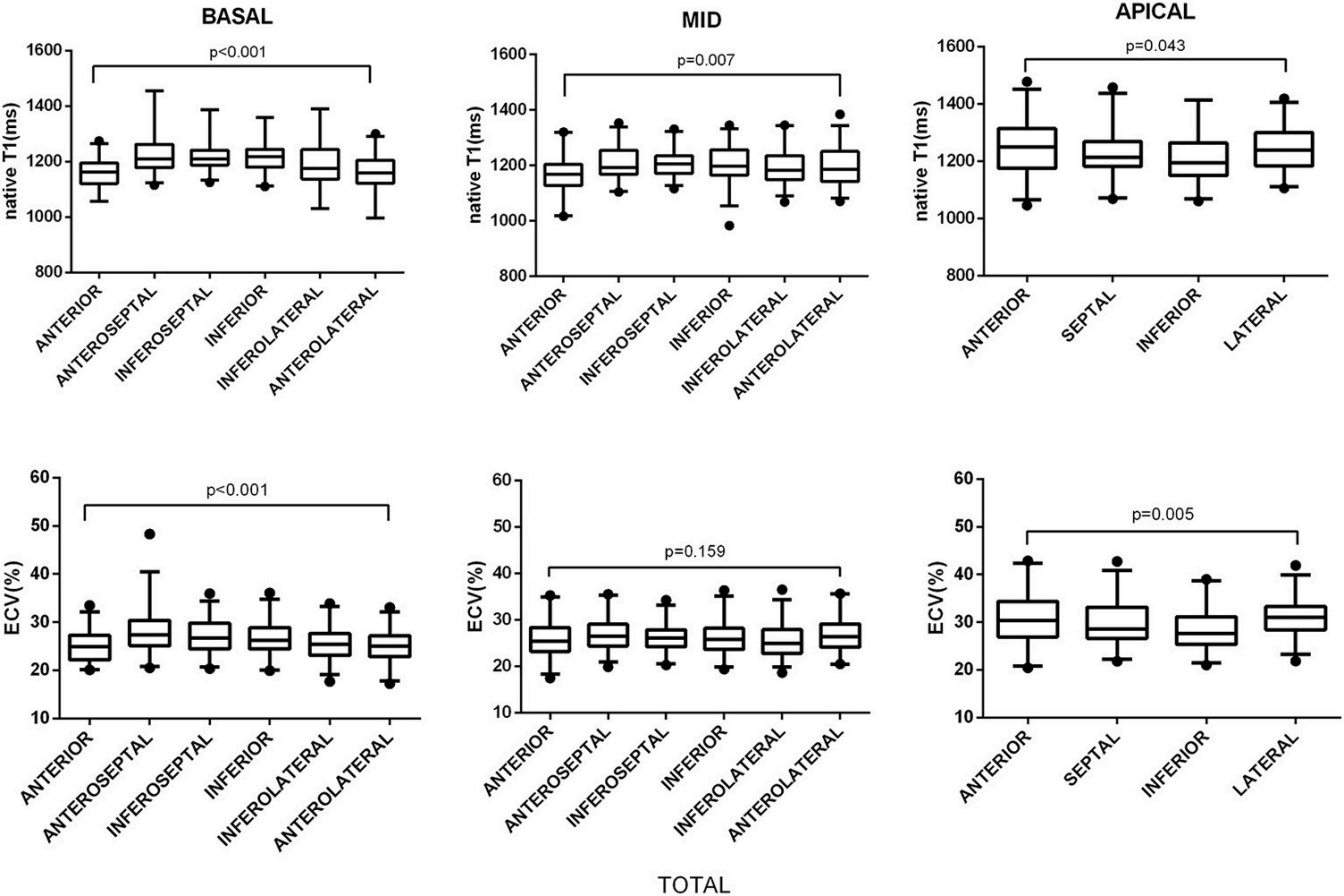
Segmental variations of native T1 value and ECV by slice location. The central box represents the values from the lower to the upper quartile. The middle line shows the median. The whiskers range from the minimum to the maximum value excluding outliers, which are shown as dots. Differences in native T1 and ECV between segments were analyzed using the Kruskal–Wallis test.

All segmental T1 and ECV are shown in the bull's-eye figure, according to the AHA 16 segment model (Figure 1). For the basal slice, there was a significant difference in T1 value and ECV among the segments (Both *P* < 0.001). Septal segments had the highest T1 and ECV values. Anteriorlateral walls had the lowest T1 and ECV values. Significant segmental inhomogeneity of T1 or ECV was found in the mid- and apical-slices (*P* < 0.05) except for ECV of the mid slice, shown in Figure 2.

### Inter- and intra-observer variability

Inter- and intra-observer variability of native T1 and ECV were tested. Both mean global T1 value and ECV showed good reproducibility (ICCs > 0.8), while reproducibility in segmental T1 and ECV was moderate (ICCs > 0.6) (data in Supplementary Tables 1, 2).

## Discussion

Our study investigated LV T1 and ECV values in a cohort of healthy Chinese adults using a CMR T1 mapping technique. We compared and showed that there are significant differences in myocardial native T1 and ECV values by gender or age subgroups. No other physiological parameters correlated significantly with native LV myocardial T1 or ECV. We also observed segmental variations across the LV for both native myocardial T1 and ECV.

Native myocardial T1 and ECV in healthy populations has been reported in various populations (Supplementary Table 3), however, most of these studies were small and focused on patients with European heritage. Supplementary Table 3 displays previously reported results of myocardial T1 and/or ECV in various populations and at various magnetic field strengths. In our present study, mean LV myocardial native T1 value was 1,202 ± 45 ms, which is similar to the result shown by Liu et al. (2014; 1,232 ± 51 ms). Native T1 value in our study was higher than reported by Dabir et al. (2014; 1,052 ± 23 ms). Mean myocardial T1 value (1158.7 ms) reported by von Knobelsdorff-Brenkenhoff et al. from another European group was also lower than our present result (von Knobelsdorff-Brenkenhoff et al., 2013). However, other studies reported higher myocardial T1 value than ours (Kawel et al. in the USA: 1,286 ± 59 ms; Lee et al. in the USA: 1,315 ± 39 ms; Lee et al., 2011; Kawel et al., 2012a). These variations could reflect influences from different scanners, populations, sample size, age group, among other influences. In contrast to myocardial native T1 variation, ECV across various studies was similar (Supplementary Table 3). Reported ECV ranged from 25 to 27% in healthy volunteers even in the presence of different magnetic field strengths (Raman et al., 2013). This finding has been confirmed by a study that demonstrated that ECV measured at 1.5T or 3.0T was very similar (Kawel et al., 2012a). ECV might be more robust to compare across different centers compared with native T1.

Analysis of gender differences in myocardial native T1 and ECV gave inconsistent results in previous studies (Sado et al., 2012; Ugander et al., 2012; Piechnik et al., 2013; von Knobelsdorff-Brenkenhoff et al., 2013; Dabir et al., 2014; Liu et al., 2014; Rauhalammi et al., 2016). In our study, gender difference was found in both native T1 and ECV, with females having higher native T1 and ECV values than males. This finding is consistent with some previous studies. Piechnik et al. reported native T1 measured by shMOLLI at 1.5T in 342 healthy subjects and showed that females had higher native T1 than males (Piechnik et al., 2013). In the MESA study, higher native T1 values were found in females vs. males in a multi-ethnic middle to older aged population (Liu et al., 2013). However, work by Liu et al. and von Knobelsdorff-Brenkenhoff et al. did not show gender differences in native T1 or ECV (Liu et al., 2013; von Knobelsdorff-Brenkenhoff et al., 2013). In Liu's study, the number of male and female subjects was not equal (male:female = 38:54), possibly affecting the comparison. The mechanism of gender differences in native T1 value and ECV remains unknown. In an autopsy study of gender and aging (Olivetti et al., 1995), the authors elegantly demonstrated that the myocytes number, size, and the interstitium remained fairly constant throughout life in females, but there was a significant loss of myocyte number and thus an increase of myocardial size, possibly through myocyte fusion and a loss of interstitium in males. Thus, a higher native T1 and ECV value might reflect the larger myocardial interstitium in females. Further research is needed to understand the relationship of interstitial fibrosis and diastolic heart failure in women.

In our study, we found a significant correlation between age and ECV. The relationship between age and native T1 or ECV were conflicting in previous studies (Liu et al., 2013; Piechnik et al., 2013; von Knobelsdorff-Brenkenhoff et al., 2013; Dabir et al., 2014; Rauhalammi et al., 2016). ECV, which might be a more consistent parameter to evaluate myocardial fibrosis, was significantly associated with age in the MESA study and in a study by Ugander et al. (Ugander et al., 2012; Liu et al., 2013). While other studies showed no significant age dependency of ECV (Sado et al., 2012; Liu et al., 2013; Dabir et al., 2014).

In the present study, no association between LV myocardial strain and native T1 or ECV was found. In addition, LAVmaxi did not correlate with either native T1 or ECV. This could be explained by small differences of T1 or ECV in healthy subjects. In studies with cardiovascular diseases, ECV was found to be significantly correlated with ventricular function (Ugander et al., 2012; Dusenbery et al., 2014).

In previous studies, segmental variations of myocardial T1 value or ECV were observed. von Knobelsdorff-Brenkenhoff et al. reported myocardial native T1 increased from basal slice to apex. This phenomenon was explained by partial volume effect of image acquisition at the apex (von Knobelsdorff-Brenkenhoff et al., 2013; Rogers and Puntmann, 2014). The result of our study is consistent with the result of Knobelsdorff-Brenkenhoff et al.'s study. In our study, native T1 and ECV showed incrementally increased from the basal to apical slice. When analyzing segmental variation, the basal, mid, or apical slice showed significantly heterogeneous T1 and ECV. The septum had the highest T1 and ECV. This result is consistent with previous studies (Kawel et al., 2012b; von Knobelsdorff-Brenkenhoff et al., 2013). Messroghli et al. did not find segmental variation of T1 value, but their study included only nine healthy subjects (Messroghli et al., 2006). The mechanism of native T1 or ECV heterogeneity in different segments is not clear. Motion artifact or partial volume effect was previously thought to be the major causes (Rogers and Puntmann, 2014), as the septum moves less than the lateral wall. Inherent myocardial composition difference could be one of the reasons. Varied myocardial fibrotic remodeling was found in variable disease states, e.g., mid-wall septal LGE is common in dilated cardiomyopathy, hypertrophic cardiomyopathy and pulmonary hypertension (Shehata et al., 2011). In patients with pulmonary hypertension, native T1 in the septum was significantly higher than normal control (Spruijt et al., 2016). These studies suggested that the septum may be an area of early remodeling during pathophysiological processes. Therefore, analysis of segmental native T1 or ECV could have potential pathophysiological significance.

## Limitations

The sample size of the present study is small. Large sample size studies are necessary to explore physiological changes of interstitial LV myocardial fibrosis evaluated by the T1 mapping technique. In addition, hormone and other serum biomarker were not available. Correlations between native T1 and ECV and biomarkers also need further exploration. Finally, T1 and ECV normal reference are magnetic field strength-, sequence-, and vendor-dependent. Therefore, the reference range in the present study provided only a reference for the same field strength and scanner.

## Conclusion

In summary, the present study showed that gender and age have different impact on myocardial interstitial fibrosis in a healthy adult Chinese population. Segmental variation of myocardial interstitial fibrosis was also observed. This finding may help us to understand physiological changes of myocardial interstitial fibrosis in the Chinese population.

## Author contributions

YD participated in the study design, contributed to data analysis and interpretation, performed the statistical analysis, and drafted the manuscript; DY carried out data acquisition and analyzed the imaging data and contributed to study design and review of the manuscript and he contributed equally to this manuscript; YC is the supervisor of this study and contributed to study design, contributed to preparation, editing and review of the final manuscript; YH contributed to study design and helped revise the manuscript; KW and HL analyzed the imaging data; JS and WC carried out subject scanning and performed data analysis and interpretation; GA and XZ provided technical support in carrying out the T1 mapping study. All the authors read, helped to revise and approved the final manuscript.

### Conflict of interest statement

The authors declare that the research was conducted in the absence of any commercial or financial relationships that could be construed as a potential conflict of interest.
