# Supplementary material for: Age and Gender Impact the Measurement of Myocardial Interstitial Fibrosis in a Healthy Adult Chinese Population: A Cardiac Magnetic Resonance Study
Source: Front Physiol. 2018 Mar 6;9:140. doi: 10.3389/fphys.2018.00140 (PMC5845542; doi:10.3389/fphys.2018.00140)
Supplement: Supplementary file 1 [file Table1.DOCX]

**Supplementary Table 1 inter-observer and intra-observer variability for native T1**

|  |  | Intra-observer | | | Inter-observer | | |
| --- | --- | --- | --- | --- | --- | --- | --- |
|  |  | Mean bias±SD(ms) | ICC | COV(%) | Mean bias±SD(ms) | ICC | COV(%) |
|  | basal | -7.7±50.0 | 0.88 | 1.53 | 0.6±36.9 | 0.94 | 1.07 |
| Total | mid | -0.5±28.0 | 0.89 | 0.83 | -1.2±33.6 | 0.84 | 0.99 |
|  | apical | 11.1±54.4 | 0.95 | 1.66 | -2.6±54.2 | 0.95 | 1.54 |
|  |  |  |  |  |  |  |  |
|  | anterior | -5.0±38.9 | 0.93 | 1.22 | -17.4±61.0 | 0.87 | 2.12 |
|  | anteroseptal | 2.6±52.9 | 0.84 | 1.53 | -11.5±48.5 | 0.87 | 1.54 |
| Basal | inferoseptal | -5.6±39.3 | 0.84 | 1.18 | 11.3±44.2 | 0.75 | 1.45 |
|  | inferior | -10.9±89.0 | 0.89 | 2.54 | -5.6±100.4 | 0.88 | 2.78 |
|  | inferolateral | -8.4±82.0 | 0.87 | 2.45 | 9.6±68.3 | 0.90 | 2.08 |
|  | anterolateral | -13.8±34.2 | 0.97 | 1.33 | 13.5±52.4 | 0.93 | 1.78 |
|  |  |  |  |  |  |  |  |
|  | anterior | -9.7±62.9 | 0.88 | 2.01 | -12.9±55.9 | 0.92 | 1.86 |
|  | anteroseptal | -6.1±37.0 | 0.89 | 1.14 | 0.3±42.1 | 0.79 | 1.23 |
| Mid | inferoseptal | 0.6±21.6 | 0.68 | 0.63 | -2.4±26.0 | 0.66 | 1.16 |
|  | inferior | 1.1±47.3 | 0.68 | 2.03 | -2.5±43.4 | 0.71 | 2.19 |
|  | inferolateral | 8.7±61.1 | 0.76 | 1.89 | -2.6±58.9 | 0.77 | 1.76 |
|  | anterolateral | 7.2±86.7 | 0.67 | 2.61 | 0.4±65.4 | 0.84 | 1.95 |
|  |  |  |  |  |  |  |  |
|  | anterior | 21.8±113 | 0.92 | 3.38 | -8.2±97.5 | 0.95 | 2.77 |
| Apical | septal | 6.3±84.8 | 0.86 | 2.41 | 3.1±56.3 | 0.94 | 1.60 |
|  | inferior | 15.5±62.2 | 0.92 | 2.01 | -4.5±92 | 0.81 | 2.67 |
|  | lateral | -2.1±52.9 | 0.94 | 1.49 | 5.5±93.1 | 0.86 | 2.63 |

ICC:inter-class correlation coefficient; COV: coefficient of variation; SD: standard deviation
