# Supplementary material for: Age and Gender Impact the Measurement of Myocardial Interstitial Fibrosis in a Healthy Adult Chinese Population: A Cardiac Magnetic Resonance Study
Source: Front Physiol. 2018 Mar 6;9:140. doi: 10.3389/fphys.2018.00140 (PMC5845542; doi:10.3389/fphys.2018.00140)
Supplement: Supplementary file 2 [file Table2.DOCX]

**Supplementary Table 2 inter-observer and intra-observer variability for ECV**

|  |  | Intra-observer | | | Inter-observer | | |
| --- | --- | --- | --- | --- | --- | --- | --- |
|  |  | Mean bias ±SD(%) | ICC | COV(%) | Mean bias ±SD(%) | ICC | COV(%) |
|  | basal | 0.13±1.19 | 0.92 | 1.70 | 0.02±1.76 | 0.82 | 2.46 |
| Total | mid | -0.23±1.52 | 0.90 | 2.13 | 0.36±1.67 | 0.89 | 2.48 |
|  | apical | 0.00±3.30 | 0.88 | 3.91 | 0.20±2.60 | 0.92 | 3.19 |
|  |  |  |  |  |  |  |  |
|  | anterior | 0.51±1.86 | 0.85 | 3.04 | -0.29±1.53 | 0.88 | 2.36 |
|  | anteroseptal | 0.17±1.74 | 0.91 | 2.40 | 0.00±3.20 | 0.61 | 4.33 |
| Basal | inferoseptal | -0.40±2.10 | 0.85 | 3.32 | 0.50±2.60 | 0.76 | 3.99 |
|  | inferior | 0.50±2.30 | 0.85 | 3.33 | -0.40±2.60 | 0.85 | 3.65 |
|  | inferolateral | 0.00±3.40 | 0.63 | 4.79 | 0.40±3.50 | 0.62 | 5.05 |
|  | anterolateral | 0.17±1.74 | 0.89 | 2.88 | 0.33±1.89 | 0.90 | 2.88 |
|  |  |  |  |  |  |  |  |
|  | anterior | 0.48±1.59 | 0.97 | 2.52 | 0.50±3.00 | 0.91 | 4.23 |
|  | anteroseptal | 0.15±1.58 | 0.91 | 2.21 | 0.40±2.60 | 0.71 | 3.77 |
| Mid | inferoseptal | -1.00±3.90 | 0.96 | 2.01 | 0.50±2.40 | 0.84 | 3.70 |
|  | inferior | -0.20±2.20 | 0.87 | 2.97 | 0.40±3.60 | 0.74 | 4.88 |
|  | inferolateral | -1.00±3.90 | 0.66 | 5.90 | 1.00±3.80 | 0.65 | 6.01 |
|  | anterolateral | -0.70±1.50 | 0.73 | 4.16 | 0.40±3.40 | 0.72 | 4.68 |
|  |  |  |  |  |  |  |  |
|  | anterior | -0.90±8.10 | 0.73 | 9.72 | -0.40±7.00 | 0.77 | 8.33 |
| Apical | septal | 0.10±2.20 | 0.93 | 2.71 | 0.30±2.30 | 0.90 | 3.03 |
|  | inferior | 0.20±4.00 | 0.85 | 5.00 | -0.08±1.94 | 0.97 | 2.43 |
|  | lateral | 0.50±2.00 | 0.95 | 2.65 | -0.00±5.50 | 0.66 | 6.49 |

ICC:inter-class correlation coefficient; COV: coefficient of variation; SD: standard deviation
