# Supplementary material for: Age and Gender Impact the Measurement of Myocardial Interstitial Fibrosis in a Healthy Adult Chinese Population: A Cardiac Magnetic Resonance Study
Source: Front Physiol. 2018 Mar 6;9:140. doi: 10.3389/fphys.2018.00140 (PMC5845542; doi:10.3389/fphys.2018.00140)
Supplement: Supplementary file 3 [file Table3.DOCX]

**Supplementary Table 3. Summary of T1 mapping reference values in normal subjects**

| Author | N (M:F) | Age (y) | Country or race | Sequence | Gd type & dose (mmol/kg) | Native T1 (ms) | ECV (%) | Slice(s) & thickness |
| --- | --- | --- | --- | --- | --- | --- | --- | --- |
| **3.0T** |  |  |  |  |  |  |  |  |
| Rauhalammi (2016) | 84 (43:41) | 45 ± 18 | UK | MOLLI | 0.15 Magnevist | 1154.7 ± 26.2 | 25.0 ± 2.3 | 3 × SAX full thickness |
| Dabir (2014) | 102 (53:49) | 17-83 | UK | MOLLI | 0.1, 0.15 or 0.2 Gadovist | 1052 ± 23 | 26 ± 4 | 1 × SAX full thickness |
| Liu (2014) | 92 (38:54) | 27-44 | African American | MOLLI | No contrast given | 1232 ± 51 | Not reported | 4Ch full thickness |
| Brenkenhoff (2013) | 60 (30:30) | 48 ± 17 | Germany | MOLLI | 0.2 Gadovist | 1074.0-1250.7 | Not reported | 3 × SAX full thickness |
| Puntmann (2013) | 30 (19:11) | 43 ± 9 | UK | MOLLI | 0.2 Gadobutrol | 1070 ± 55 | 27 ± 1 | 1 × SAX septal midwall |
| Kawel (2012) | 29 (9:20) | 28 ± 6 | USA | MOLLI | 0.15 Magnevist | Not reported | Not reported | 1 × SAX full thickness |
| Kawel (2012) | 24 (8:16) | 28 ± 6 | USA | MOLLI | 0.15 Magnevist | 1286 ± 59 | 27 ± 3 | 1 × SAX full thickness |
| Lee (2011) | 11 (6:5) | 36 ± 13 | USA | MOLLI | 0.15 Magnevist | 1315 ± 39 | 26.7 ± 1.0 | 1 × SAX full thickness |
| **1.5T** |  |  |  |  |  |  |  |  |
| Piechnik (2013) | 342 (173:169) | 11-69 | UK and Netherland | shMOLLI | No contrast given | 962 ± 25 | Not reported | 3 × SAX full thickness |
| Karamitsos (2013) | 36 (22:14) | 59 ± 4 | UK | shMOLLI | No contrast given | 958 ± 20 | Not reported | 1 × SAX & 4Ch full thickness |
| Sado (2012) | 81 (42:39) | 44 ± 17 | UK | FLASH IR | 0.1 Dotarem | Not reported | 25.3 ± 3.5 | 2 × SAX septum |
| Ugander (2012) | 60 (31:29) | 49 ± 17 | USA | MOLLI | 0.15-0.2 Magnevist | Not reported | 27 ± 3 | 1 × SAX & 4Ch full thickness |
| Messroghli (2006) | 9 (3:6) | 33.1 ± 8.5 | UK | MOLLI | No contrast given | 979 ± 54 | Not reported | 3 × SAX full thickness |

N: number; M: male; F: female; SAX: short axis
